# A Systematic Review of Candidate miRNAs, Its Targeted Genes and Pathways in Chronic Myeloid Leukemia–An Integrated Bioinformatical Analysis

**DOI:** 10.3389/fonc.2022.848199

**Published:** 2022-03-07

**Authors:** Marjanu Hikmah Elias, Syarifah Faezah Syed Mohamad, Nazefah Abdul Hamid

**Affiliations:** ^1^Faculty of Medicine and Health Sciences, Universiti Sains Islam Malaysia, Nilai, Malaysia; ^2^Faculty of Applied Sciences, Universiti Teknologi MARA Cawangan Pahang, Jengka, Malaysia

**Keywords:** chronic myeloid leukemia, genes, pathways, microRNA, BCR-ABL1

## Abstract

Chronic myeloid leukaemia is blood cancer due to a reciprocal translocation, resulting in a BCR-ABL1 oncogene. Although tyrosine kinase inhibitors have been successfully used to treat CML, there are still cases of resistance. The resistance occurred mainly due to the mutation in the tyrosine kinase domain of the BCR-ABL1 gene. However, there are still many cases with unknown causes of resistance as the etiopathology of CML are not fully understood. Thus, it is crucial to figure out the complete pathogenesis of CML, and miRNA can be one of the essential pathogeneses. The objective of this study was to systematically review the literature on miRNAs that were differentially expressed in CML cases. Their target genes and downstream genes were also explored. An electronic search was performed *via* PubMed, Scopus, EBSCOhost MEDLINE, and Science Direct. The following MeSH (Medical Subject Heading) terms were used: chronic myeloid leukaemia, genes and microRNAs in the title or abstract. From 806 studies retrieved from the search, only clinical studies with *in-vitro* experimental evidence on the target genes of the studied miRNAs in CML cells were included. Two independent reviewers independently scrutinised the titles and abstracts before examining the eligibility of studies that met the inclusion criteria. Study design, sample size, sampling type, and the molecular method used were identified for each study. The pooled miRNAs were analysed using DIANA tools, and target genes were analysed with DAVID, STRING and Cytoscape MCODE. Fourteen original research articles on miRNAs in CML were included, 26 validated downstream genes and 187 predicted target genes were analysed and clustered into 7 clusters. Through GO analysis, miRNAs’ target genes were localised throughout the cells, including the extracellular region, cytosol, and nucleus. Those genes are involved in various pathways that regulate genomic instability, proliferation, apoptosis, cell cycle, differentiation, and migration of CML cells.

## Introduction

Chronic myeloid leukaemia (CML) is a proliferative disorder of pluripotent stem cells. CML is linked to a specific genetic disorder involving *BCR* and *ABL1* gene translocation, resulting in the Philadelphia chromosome. *BCR-ABL1* fusion gene encodes an active tyrosine kinase BCR-ABL which activates several molecular pathways that cause abnormal cell adhesion, increase cell proliferation, and inhibit apoptosis. Nevertheless, tyrosine kinase plays an essential role in many signalling cascades, including biological processes such as cell growth, differentiation, metabolism, and apoptosis (1). Numbers of studies have revealed the mechanisms of CML pathogenesis which involved several key signalling pathways, including the MAPK, JAK-STAT, PI3K/AKT, EGFR, ERBB, TGF-β and tumour protein p53 pathways (2–4).

Thus, specific treatment for CML has been developed by targeting the *BCR-ABL1* gene. The first molecularly targeted therapy, Imatinib, is a small molecule known as tyrosine kinase inhibitor (TKI) that directly targets *BCR-ABL1* tyrosine kinase activity. Despite the success of Imatinib as the front-line therapy for CML, there were reports on drug resistance that is primarily due to the presence of mutations in the *BCR-ABL* tyrosine kinase domain (TKD) (5–7). Tyrosine kinase inhibitors are not able to fully prevent the progression of CML cells with BCR-ABL TKD mutated CML cells. Therefore, research into alternative treatments for CML remains clinically essential. In recent years, microRNA (miRNA) has been widely studied in human malignancies and chemical compounds. They have received widespread attention as essential regulators of gene expression in leukemogenesis and are linked to resistance to *BCR-ABL1* TKIs (3, 8).

MiRNA is a short, non-coding RNA that regulates gene expression at the post-transcriptional level. It inhibits translation by binding to the 3’untranslated (3’UTR) region of specific target mRNA. MiRNA has been linked to disease pathogenesis in CML and is known to play an essential role in tumorigenesis (9). MiRNA’s roles and functions have been highlighted in several studies in a variety of circumstances and scenarios. For instance, miRNAs expression profiles were used as biomarkers and therapeutic tools (10–12). Their expression differences were used in several studies to improve response prediction in diseases, particularly CML. Aberrant miRNA expression is linked to stem cell survival, cell renewal and sensitivity or resistance to TKI therapy, all of which contribute to disease progression (1, 13, 14). Additionally, miRNAs have been discovered to affect genes in signalling pathways involved in cell proliferation, apoptosis, leukemogenesis, and tumour suppression (15–17). Hence, the current study aims to identify and screen differentially expressed miRNAs in CML patients from previous literatures. The application of integrated bioinformatics analysis is essential in predicting miRNA target genes, gene ontology and pathways, and protein-protein interaction networks. Findings from this study will help researchers to better understand the role of miRNA in CML pathogenesis and treatment resistance.

## Methods

### Search Strategy

A comprehensive search of information was done using PubMed, Scopus, EBSCOhost MEDLINE, and Science Direct to identify relevant research publications with an unlimited starting publication date until 1st April 2021. The Medical Subject Heading (MeSH) terms like chronic myeloid leukemia, genes and microRNAs were used as the keywords in the title or abstract. The search strategy involved a combination (“AND”) of the following two sets of keywords (1): “chronic myelo* leukemia” OR CML OR “BCR*ABL*positive” and (2) mi*RNA. Synonyms for keywords were generated through MeSH terms from the Cochrane Library. Additional text terms were discovered by reviewing collected review articles. Additional references were discovered from the bibliographies of the retrieved studies.

### Inclusion Criteria

Case-control and prospective observational studies with abstracts investigating the differentially expressed miRNAs on Philadelphia chromosome-positive chronic myeloid leukemia patients in chronic, accelerated, or blast phases were included. In addition, only clinical studies that have further *in vitro* experimental evidence on the target genes of the studied miRNAs in CML cells were included in this review. Due to limited resources, only manuscripts written in English were included.

### Exclusion Criteria

Publications that did not have primary data, such as editorials, case reports, conference proceedings, and narrative review articles, were excluded. In silico, *in vitro*, and *in vivo* studies were excluded. The review focus on the outcome of the differentially expressed miRNAs in CML patients. Therefore, studies that involved responses toward tyrosine kinase inhibitors treatment or any other intervention studies on a new treatment for CML patients were excluded from consideration. These selection criteria were used to achieve the objective of this systematic review in determining the typical miRNA expression signature in CML patients, the miRNAs target genes, and related pathways that could potentially be involved in the pathogenesis of CML.

### Screening of Articles for Eligibility

**Figure 1:**
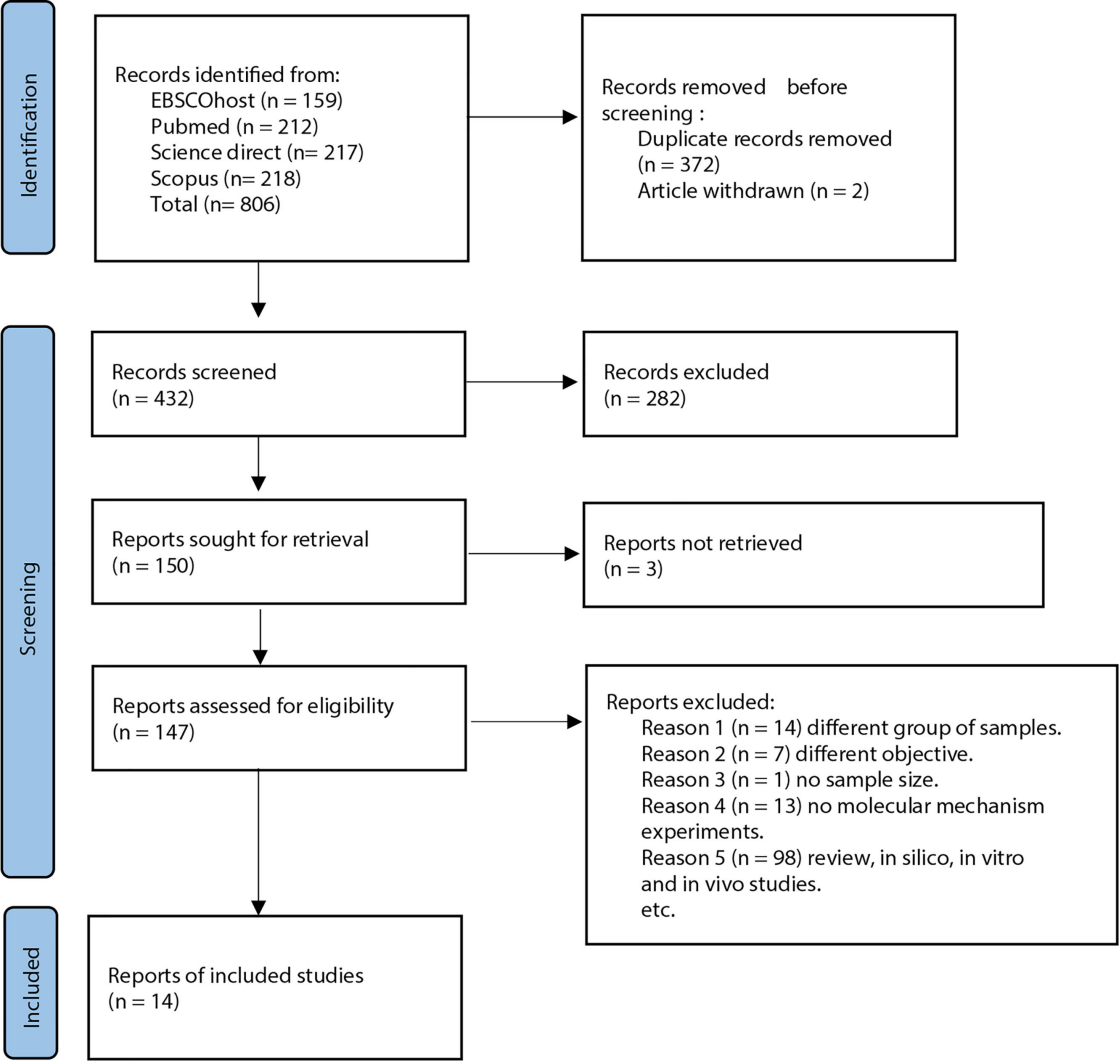
PRISMA flow diagram for studies selection in this systematic review.

Articles retrieved from all resources were screened in three phases. All articles with titles that did not match the inclusion criteria were excluded, and duplicates were removed in the first phase. The abstracts of the remaining articles were screened, and any articles that did not meet the inclusion criteria were excluded in the second phase. Finally, the full texts of the remaining articles were read and assessed thoroughly. Systematic reviews, meta-analyses, and articles that did not meet the inclusion criteria were excluded in this third phase. All the authors were involved in the screening, selection, and data extraction phase. Any differences in opinions were resolved by discussion between the authors. All data extraction was performed independently using a data collection form to standardize the data collection, and records on reasons for rejection were kept. **Figure 1** shows the flow chart that summarizes the article selection process and the reasons for article exclusion.

### Data Extraction and Study Quality

Data were extracted from the studies that fulfilled inclusion criteria. Data collected from these studies include (1) author name (2), study design (3), study objective (4), study population (5), type of sample used (6), method used in gene expression analysis and experiments performed to validate their targeted genes (7), results (upregulated and downregulated miRNAs, their validated targeted genes and downstream effected genes), and (8) conclusion. The extracted details are listed in **Table 1**.

**Table 1:** Summary of selected studies.

Title (References)	Study design	Samples(Type)	Methodology		Findings	
			Gene expression analysis	Target gene analysis	MiRNA (regulation)	Targeted gene
MiR-152-3p promotes the development of chronic myeloid leukemia by inhibiting p27 (18).	Case-control study	CML (n=40) Controls (n=40) (BM)	qPCR	Luciferase assay	miR-152-3p (upregulated)	*CDKN1B*
MiR-362-5p promotes the malignancy of chronic myelocytic leukaemia *via* downregulation of GADD45α (19)	Case-control study	CML (n=40) Controls (n=26) (PB)	qPCR	Luciferase assay	miR-362-5p (upregulated)	*GADD45A*
miR-140-5p induces cell apoptosis and decreases Warburg effect in chronic myeloid leukemia by targeting SIX1 (20)	Case-control study	CML (n=30) Controls (n=30) (PB)	qPCR	Luciferase assay	miR-140-5p (downregulated)	*SIX1*
MiRNA-409-5p dysregulation promotes imatinib resistance and disease progression in children with chronic myeloid leukemia (21)	Case-control study	CML (n=42) Controls (n=40) (PB)	qPCR	Luciferase assay	miR-409-5p (downregulated)	*NUP43*
MiR-570 inhibits cell proliferation and glucose metabolism by targeting IRS1 and IRS2 in human chronic myelogenous leukemia (22)	Cross sectional study	CML (n=15) (PB)	qPCR	Luciferase assay	miR-570-3p (downregulated)	*IRS1* *IRS2*
MicroRNA-320a acts as a tumor suppressor by targeting BCR/ABL oncogene in chronic myeloid Leukemia (23)	Case-control study	CML (n=90) Controls (n=90) (BM)	qPCR	Luciferase assay	miR-320a (downregulated)	*BCR-ABL1*
Restoration of miR-424 suppresses BCR–ABL activity and sensitizes CML cells to imatinib treatment (24)	Case-control study	CML (n=18) Controls (n=10) (PB)	qPCR	Luciferase assay	miR-424-5p (downregulated)	*BCR-ABL1*
The malignancy suppression role of miR-23a by targeting the BCR/ABL oncogene in chromic myeloid leukemia (25)	Case-control study	CML (n=79) Controls (n=25) (BM)	qPCR	Luciferase assay	miR-23a-3p (downregulated)	*BCR-ABL1*
Low Expression of miR-196b Enhances the Expression of *BCR-ABL1* and HOXA9 Oncogenes in Chronic Myeloid Leukemogenesis (26)	Case-control study	CML (n=16) Controls (n=10) (BM)	qPCR	Luciferase assay	miR-196b (downregulated)	*BCR-ABL1* *HOXA9*
Decreased microRNA-30a levels are associated with enhanced ABL1 and *BCR-ABL1* expression in chronic myeloid leukemia (27)	Case-control study	CML (n=16) Controls (n=10) (BM)	qPCR	Luciferase assay	miR-30a (downregulated)	*BCR-ABL1*
miR-29b suppresses CML cell proliferation and induces apoptosis *via* regulation of BCR/ABL1 protein (28)	Case-control study	CML (n=5) Controls (n=3) (BM)	qPCR	Luciferase assay	miR-29b (downregulated)	*BCR-ABL1*
Targeting of the signal transducer Smo links microRNA-326 to the oncogenic Hedgehog pathway in CD341 CML stem/progenitor cells (29)	Case-control study	CML (n=6) Controls (n=4) (BM)	qPCR	Luciferase assay	miR-326 (downregulated)	*SMO*
BCR-ABL mediated repression of miR-223 results in the activation of MEF2C and PTBP2 in chronic myeloid leukemia (30)	Cross sectional study	CML (n=35) (PB)	qPCR	Luciferase assay	miR-223 (downregulated)	*MEF2C* *PTBP2*
Down-Regulation of hsa-miR-10a in Chronic Myeloid Leukemia CD34+ Cells Increases USF2-Mediated Cell Growth (31)	Case-control study	CML (n=6) Controls (n=6) (BM)	qPCR	Luciferase assay	miR-10a (downregulated)	*USF*

The analysis details of each report were discussed thoroughly among the reviewers to assess the quality of each study. The authors focused on the reported list of miRNAs as well as their targeted genes. Bias was excluded by adhering to the inclusion criteria. Pairs of reviewers with adequate reliability worked independently to determine the validity of each study.

### Prediction of miRNA Target Genes

The miRNAs listed were further analysed using four different bioinformatics tools that include 1) DIANA-microT web server v5.0 with MiTG scores being set at more than 0.95, 2) TargetScan release 7.2 with Cumulative weighted context++ score of more than -0.5, 3) miRDB with a target score more than 90, and 4) mirDIP v5.0.2.3 with score class set at “very high”. Genes that are predicted by more than two bioinformatics tools were selected for further analysis.

### Gene Ontology and Pathway Enrichment Analysis

Two groups of analysis that include 1) a group of validated target genes and downstream genes of the miRNAs extracted from the studies and 2) a group of predicted target genes of the reported miRNAs was done. These two groups of genes were analyzed using Database for Annotation, Visualization, and Integrated Discovery (DAVID). DAVID was performed to determine the cluster of genes that displayed significant functional annotation enrichment related to CML’s pathogenesis. The contribution of genes in the pathway related to CML was based on the Kyoto Encyclopedia of Genes and Genomes (KEGG) pathway, the Biological Biochemical Image database (BBID), BIOCARTA pathway database, and Reactome.

### Protein–Protein Interaction (PPI) Network

The targeted genes were further analyzed at the protein level using protein-protein interaction network functional enrichment analysis through STRING (Protein-Protein Interaction Network Functional Enrichment Analysis) (https://string-db.org/). Results from STRING were further analyzed using Cytoscape to visualize molecular interaction networks and integrating gene expression profiles to identify clusters of protein interaction that are highly related to the pathogenesis of CML. The gene interaction relationship was downloaded in the “TSV” format file and was imported into the Cytoscape software (http://www.cytoscape.org/) for further analysis and clustering. The Cytoscape MCODE plug-in was employed to perform module analysis of the target network and protein clustering. The module selection criteria were as follows: degree cutoff = 2, node score cutoff = 0.2, node density cutoff = 0.1, K-score = 2, and max depth = 100. The list of genes in the cluster was then analyzed again using DAVID for significantly enriched ontology terms.

## Results

A total of 806 potentially relevant titles were identified from the database search. EndNote X9 software by Clarivate Analytics (Philadelphia, USA) was used as the reference manager. Upon filtering the titles, 372 articles were identified as duplicates, and two other articles were withdrawn from publication. A total of 432 articles were retrieved for abstract reviewing. Upon screening titles and abstracts, 263 articles were removed, resulting in the selection of 159 potentially relevant articles for full-text review. However, three articles could not be retrieved as the full text are not in English. Then, 147 potentially relevant articles’ full text was thoroughly reviewed, and 130 articles were eliminated based on our inclusion and exclusion criteria. Finally, 17 articles were selected to be included in the present systematic review. All studies were original research articles published between the year 2008 to 2019. Homogeneity of the selected studies was ensured by adhering to the defined inclusion and exclusion criteria to prevent sampling bias. Notably, all the studies performed real-time polymerase chain reaction (qPCR) for miRNA expression analysis. A confirmatory method was done to validate the miRNA binding site on their selected genes. Sample sizes for each study varied from 8 to 180 samples for miRNA expression analysis. The characteristics of these studies are highlighted in **Table 1**.

### Patient Recruitment and Sampling

Samples collections were described briefly in most of the study. Six studies collected peripheral blood samples, while eight studies collected bone marrow tissues (**Table 1**). The inclusion criteria listed in the study by Nie et al. (20) include diagnosis *via* bone marrow morphology, immunology, molecular biology, and cytogenetic result, with no chemotherapy treatment before the specimen collection (20). In most studies, samples were collected at diagnosis; thus, most patients were in the chronic phase during sample collection. However, the study by Babashah et al. (29) collected samples from CML patients in the blast crisis phase at diagnosis (29).

### Effect of miRNA on Cells

The effect of miRNA on cells, including proliferation, apoptosis, cell cycle, migration, and invasion, were adequately reported in the studies. Among miR-409-5p, miR-424, miR-29b, miR-570, miR-320a, miR-23a, miR-196b, miR-30a, miR-326, miR-223, miR-10a that were downregulated in CML clinical samples, the low expression of miR-409-5p, miR-424, miR-29b miRNAs in CML cells were reported to be the cause of the increase in CML cell viability. The low expression of miR-570, miR-320a, miR-23a, miR-196b, miR-30a, miR-326, miR-223, miR-10a in CML cells contribute to high proliferation rate. On the other hand, overexpression of miR-152-3p, miR-362-5p were reported in CML samples and from the functional analysis done, these miRNAs contributed to the increase in proliferation rate. In the cell cycle analysis, the overexpression of miR-152-3p was reported to reduce the percentage of cells in G0/G1 phase when compared with G2SM phase. However, overexpression of miRNA-409-5p, miR-362-5p, miRNA-196b, miR-30a, miR-29b arrested cell cycle in G0/G1 phase and S phase (21). Thus, downregulation of miRNA-409-5p, miR-362-5p, miRNA-196b, miR-30a, miR-29b increase the cell cycle activities in CML cells.

From the selected studies, overexpression of miRNAs like miR-140-5p, miR-320a, miR-570 induced apoptosis, but overexpression of miRNAs like miR-362-5p reduced apoptosis. Thus, in CML cells, a high level of miR-362-5p and a low level of miR-140-5p, miR-320a, miR-570 contribute to low apoptosis activities. Overexpression of miR-320a was proved in the *in vitro* studies to inhibit CML cell migration and invasion, but overexpression of miR-362-5p increased CML cell migration and invasion. Therefore, in CML cells, upregulation of miR-362-5p and downregulation of miR-320a were proposed in cell migration and invasion pathways. Furthermore, synthetic overexpression of miR-570 suppresses glucose metabolism and reduced ATP generation in CML cells. Thus, in clinical samples, downregulation of miR-570 increases glucose metabolism and ATP generation, producing high available energy for cell growth (22).

### Targeted Gene Validation

The targeted gene validation of all miRNAs was adequately reported in all studies by co-transfecting cell lines with the targeted gene 3’-UTR reporter vector and miRNA mimic. In all studies, miRNA mimics used in the luciferase assay reveal decreased luciferase activity in wild-type targeted gene 3’UTR, suggesting that each of the miRNAs could bind to their respected genes. Mutant-type targeted gene 3’UTR did not show significant changes in luciferase activities after miRNA mimic transfection in all studies, suggesting a specific target of the miRNAs.

### Effect of miRNAs in the Expression of Downstream Genes

Some studies also reported on the downstream genes that are differently expressed related to the expression changes of their studied miRNA. Overexpression of miR-140-5p was reported to increase BAX protein expression indirectly but decreased the BCL2 protein expression *via* SIX1 in CML cells (20). Overexpression of miRNA-409-5p in CML cells indirectly leads to downregulation of NUP43, leading to downstream downregulation of PCNA, c-Myc and cyclin D1 protein (21). The expression of genes associated with glucose metabolism, namely PGC1α, PCK1 and ABCA1 proteins, were indirectly suppressed by miR-570 overexpression *via* IRS1 and IRS2 (22). Inhibition of miR-362-5p indirectly increased P38 and JNK activity in CML cells *via* GADD45A (19). MiR-320a was reported to regulate the phosphorylation of PI3K, AKT and NF-κ B *via* BCR-ABL (23). Expression of p-Crkl and p-STAT5 was reduced in the presence of miR-424 through BCR-ABL (1).

Overexpression of miR-23a resulted in lower expression of PI3K, Akt and MMP-9, which are the downstream target of BCR-ABL (25). Overexpression miR-326 indirectly downregulates SMO expression, leading to downregulation of Bcl2 expression in CML cells (29). Significant downregulation of the survival gene Bcl-xL was reported to be associated with down-regulation of MEF2C and PTBP2 due to overexpression of miR-223-3p (30). Overexpression of miR-29b led to a significant increase in BCR-ABL expression that upregulates p21 and p27 expression in CML cells (28).

### Gene Ontology Analysis of the Downstream Genes

A total of 26 downstream genes that were affected by the miRNAs was extracted from all the studies. The functions and pathway enrichment of these genes were analyzed using DAVID (https://david.ncifcrf.gov/home.jsp). A p-value of <0.05 was used as a cut-off standard. The gene listed were categorized into three functional categories of gene ontology that include biological process (BP), cellular component (CC) and molecular function (MF), as shown in **Table 2**. In the CC group, the downstream genes are enriched in the intracellular component of cells, including nucleus, cytosol, cytoplasm, nucleoplasm, and mitochondria. In the BP group, the downstream genes are enriched in the regulation of transcription, cell proliferation, apoptosis, and drug response. The downstream genes are enriched in the DNA binding, protein binding, and protein heterodimerization activities in the MF group. The complete list for gene ontology cluster is included in ‘**Data S1**’.

**Table 2:** The significantly enriched analysis of downstream genes in CML.

Term	Description	Count	p-value
MF_GO:0005515	Protein binding	20	2.18E-04
CC_GO:0005634	Nucleus	14	0.002201
CC_GO:0005829	Cytosol	12	4.13E-04
CC_GO:0005737	Cytoplasm	12	0.01868
CC_GO:0005654	Nucleoplasm	8	0.031381
BP_GO:0043524	Negative regulation of neuron apoptotic process	6	5.12E-07
BP_GO:0042493	Response to drug	6	3.02E-05
BP_GO:0008283	Cell proliferation	6	7.30E-05
MF_GO:0043565	Sequence-specific DNA binding	6	3.61E-04
BP_ GO:0045944	positive regulation of transcription from RNA polymerase II promoter	6	0.006252
BP_ GO:0006355	regulation of transcription, DNA-templated	6	0.034631
BP_ GO:0043066	negative regulation of apoptotic process	5	0.002207
MF_GO:0046982	protein heterodimerization activity	5	0.002344
BP_GO:0008284	positive regulation of cell proliferation	5	0.002408
BP_GO:0006915	apoptotic process	5	0.0048708
BP_GO:0000122	negative regulation of transcription from RNA polymerase II promoter	5	0.0112021
MF_GO:0042802	identical protein binding	5	0.012588

### Signaling Pathway Enrichment Analysis of the Downstream Genes

The miRNA targeted genes and downstream genes signalling pathway enrichment analysis were conducted using DAVID with integrated KEGG PATHWAY, BBID, BIOCARTA, and Reactome. Concerning CML pathogenesis, the genes are mainly enriched in pathways related to cancer (hsa05200), microRNAs in cancer pathway (hsa05206), Hepatitis B (hsa05161), PI3K-Akt signalling pathway (hsa04151) and many other pathways with some directly associated with apoptosis, proliferation, and cell cycle pathways as reported in **Table 3**. The complete list of pathways is included in ‘**Data S2**’.

**Table 3:** Signaling pathway enrichment analysis of downstream genes’ function in CML patients.

Pathway	Name	Count	Genes	p-value
hsa05200	Pathways in cancer	9	BCR, SMO, CCND1, MYC, BCL2, ABL1, BAX, MMP9, BCL2L1	2.79E-06
hsa05206	MicroRNAs in cancer	7	CCND1, IRS1, MYC, BCL2, ABL1, IRS2, MMP9	5.96E-05
hsa05161	Hepatitis B	6	PCNA, CCND1, MYC, BCL2, BAX, MMP9	2.67E-05
hsa04151	PI3K-Akt signaling pathway	6	CCND1, IRS1, MYC, BCL2, PCK1, BCL2L1	0.001536
h_P53Pathway	P53 Signaling Pathway	5	PCNA, CCND1, GADD45A, BCL2, BAX	7.72E-06
hsa05220	Chronic myeloid leukemia	5	BCR, CCND1, MYC, ABL1, BCL2L1	3.02E-05
hsa04152	AMPK signaling pathway	5	CCND1, IRS1, IRS2, PCK1, PPARGC1A	2.45E-04
hsa04110	Cell cycle	5	PCNA, CCND1, GADD45A, MYC, ABL1	2.53E-04
hsa04068	FoxO signaling pathway	5	CCND1, IRS1, GADD45A, IRS2, PCK1	3.41E-04
hsa05202	Transcriptional misregulation in cancer	5	MEF2C, MYC, SIX1, MMP9, BCL2L1	7.86E-04
hsa05166	HTLV-I infection	5	PCNA, CCND1, MYC, BAX, BCL2L1	0.003689
hsa05210	Colorectal cancer	4	CCND1, MYC, BCL2, BAX	5.17E-04
hsa04920	Adipocytokine signaling pathway	4	IRS1, IRS2, PCK1, PPARGC1A	7.38E-04
hsa05222	Small cell lung cancer	4	CCND1, MYC, BCL2, BCL2L1	0.001299
hsa04931	Insulin resistance	4	IRS1, IRS2, PCK1, PPARGC1A	0.002587
hsa04722	Neurotrophin signaling pathway	4	IRS1, BCL2, ABL1, BAX	0.003489
h_il2rbPathway	IL-2 Receptor Beta Chain in T cell Activation	4	IRS1, MYC, BCL2, BCL2L1	0.003877
127	Mito-stress	3	BCL2, BAX, BCL2L1	0.002915
152	Altered synaptic signalling-neurodegenerative disorders	3	BCL2, BAX, BCL2L1	0.002915
hsa05219	Bladder cancer	3	CCND1, MYC, MMP9	0.004992

### Identification of Key Candidate Genes and Pathways in the Protein–Protein Interaction Network (PPI) and Modular Analysis of the Downstream Genes

**Figure 2:**
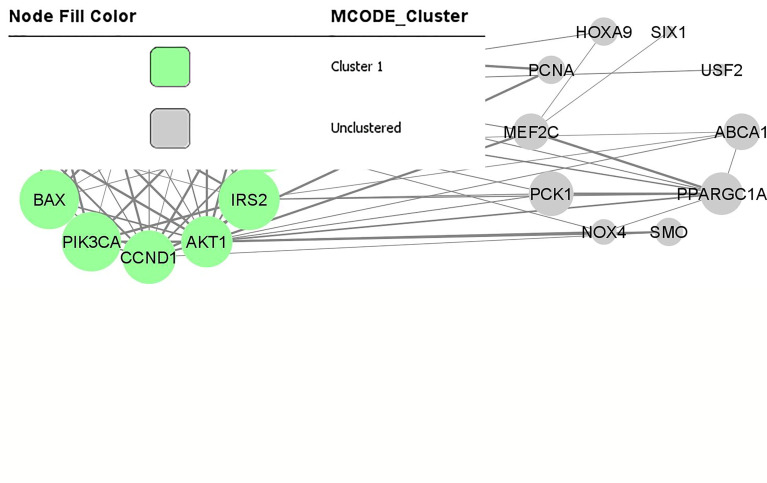
PPI network and modular analysis of downstream genes. From STRING online database analysis, a total of 26 proteins were filtered into a PPI network complex. The green nodes represent a functional annotation cluster that was identified from Cytoscape MCODE. This functional annotation clustering showed a cluster consisted of 12 proteins.

Using STRING online database (http://string-db.org), a total of 26 proteins from miRNA targets and their downstream genes were filtered into a PPI network complex, containing 22 nodes and 75 edges (**Figure 2**) with a PPI enrichment p-value is 2.22e-16. At the same time, two other proteins did not fall into the PPI network. The results were transferred from STRING to Cytoscape for further analysis. Through Cytoscape MCODE, a significant module from the PPI network complex were found. Functional annotation clustering showed that this cluster (score = 8.909) consisted of 12 nodes and 49 edges (**Figure 2**). The cluster is mainly associated with protein binding (GO:0005515) as all the 12 proteins are involved in this molecular function. Ten of the proteins can be found in the cytosol (GO:0005829), and nine are involved in cancer pathways (hsa05200). **Table 4** includes a functional annotation cluster with more than six proteins involved. The complete list for functional annotation cluster is included in ‘**Data S3**’.

**Table 4:** Functional annotation clustering on the cluster identified from downstream genes.

Term	Description	Count	p-value
GO:0005515	protein binding	12	7.56E-04
GO:0005829	cytosol	10	8.32E-06
hsa05200	Pathways in cancer	9	1.50E-08
Up_Keywords: Phosphoprotein	Phosphoprotein	9	0.029
hsa05161	Hepatitis B	8	4.92E-10
Up_Keywords: mutagenesis site	mutagenesis site	8	4.06E-05
GO:0005634	nucleus	8	0.020
hsa05210	Colorectal cancer	7	1.87E-10
hsa04068	FoxO signaling pathway	7	2.08E-08
hsa04151	PI3K-Akt signaling pathway	7	5.68E-06
Up_Keywords: Nucleus	Nucleus	7	0.037
hsa05222	Small cell lung cancer	6	1.12E-07
hsa04722	Neurotrophin signaling pathway	6	6.31E-07
h_il2rbPathway	IL-2 Receptor Beta Chain in T cell Activation	6	1.42E-06
hsa04932	Non-alcoholic fatty liver disease (NAFLD)	6	1.98E-06
GO:0043066	negative regulation of apoptotic process	6	5.77E-06
hsa05166	HTLV-I infection	6	2.54E-05
hsa05206	MicroRNAs in cancer	6	4.51E-05
GO:0005739	mitochondrion	6	6.56E-04

### Prediction of miRNA’s Targeted Genes

Apart from the downstream genes reported from the articles, target genes of the miRNAs were also identified from in silico analysis to ensure extensive coverage of miRNAs’ targets. One hundred eighty-seven target genes predicted concordantly by four different bioinformatics tools were selected for further analysis. The top fives genes are the DCP2, QKI, S1PR1, NPTN and B4GALT5. The list of genes is included in ‘**Data S4**’.

### Gene Ontology Analysis of the Predicted Target Genes

The functions and pathway enrichment of predicted target genes were analysed using DAVID (https://david.ncifcrf.gov/home.jsp). A p-value of <0.05 was used as a cut-off standard. The genes listed were categorised into three functional categories of gene ontology that include biological process (BP), cellular component (CC) and molecular function (MF), as shown in **Table 5**. In the CC group, the predicted target genes are enriched in the nucleus, proteinaceous extracellular matrix and perinuclear region of the cytoplasm. In the BP group, the genes are enriched in the regulation of transcription, extracellular matrix organisation and angiogenesis. In the MF group, the predicted target genes are enriched in the DNA binding. The list of predicted genes’ GO is included in ‘**Data S5**’.

**Table 5:** The significantly enriched analysis of predicted target genes.

Term	Description	Count	p-value
MF_GO:0005515	protein binding	106	0.010477
CC_GO:0005634	nucleus	73	0.001961
BP_GO:0006351	transcription, DNA-templated	30	0.019201
MF_GO:0003677	DNA binding	26	0.033161
BP_ GO:0045944	positive regulation of transcription from RNA polymerase II promoter	24	1.21E-04
BP_GO:0000122	negative regulation of transcription from RNA polymerase II promoter	18	9.06E-04
MF_GO:0003700	transcription factor activity, sequence-specific DNA binding	17	0.036439
MF_GO:0043565	sequence-specific DNA binding	13	0.006885
BP_GO:0030198	extracellular matrix organization	12	4.54E-06
BP_GO:0001525	angiogenesis	12	1.54E-05
CC_GO:0005578	proteinaceous extracellular matrix	12	7.45E-05
BP_GO:0006366	transcription from RNA polymerase II promoter	12	0.014271
CC_GO:0048471	perinuclear region of cytoplasm	12	0.04456
BP_GO:0030574	collagen catabolic process	10	1.39E-08
CC_GO:0005788	endoplasmic reticulum lumen	10	1.26E-04
MF_GO:0000978	RNA polymerase II core promoter proximal region sequence-specific DNA binding	10	0.010699
BP_GO:0008283	cell proliferation	10	0.01151
BP_GO:0043066	negative regulation of apoptotic process	10	0.039644

### Signaling Pathway Enrichment Analysis of the Predicted Target Genes

The predicted target genes signalling pathway enrichment analysis were conducted using DAVID with integrated KEGG PATHWAY, BBID, BIOCARTA, and Reactome. The genes were found to be mainly enriched in the PI3K-Akt signalling pathway (hsa04151), focal adhesion (hsa04510), pathways in cancer (hsa05200), and many other pathways, with each pathway, involve from 13 to six predicted target genes (**Table 6**). The complete list for predicted pathways is included in ‘**Data S6**’.

**Table 6:** Signaling pathway enrichment analysis of predicted targeted genes’ function in CML patients.

Pathway	Name	Count	p-value
hsa04151	PI3K-Akt signaling pathway	13	1.30E-05
hsa04510	Focal adhesion	10	3.13E-05
hsa05200	Pathways in cancer	10	0.003611
hsa04974	Protein digestion and absorption	8	5.43E-06
hsa04512	ECM-receptor interaction	6	6.10E-04
hsa05215	Prostate cancer	6	6.43E-04
hsa05146	Amoebiasis	6	0.001491
hsa04611	Platelet activation	6	0.003644
hsa04068	FoxO signaling pathway	6	0.004149
hsa04910	Insulin signaling pathway	5	0.024022
hsa05214	Glioma	4	0.014896
hsa05211	Renal cell carcinoma	4	0.015518
hsa05222	Small cell lung cancer	4	0.030132
hsa00512	Mucin type O-Glycan biosynthesis	3	0.025168

### Identification of Key Candidate Genes and Pathways in the Protein–Protein Interaction Network (PPI) and Modular Analysis of the Predicted Target Genes

**Figure 3:**
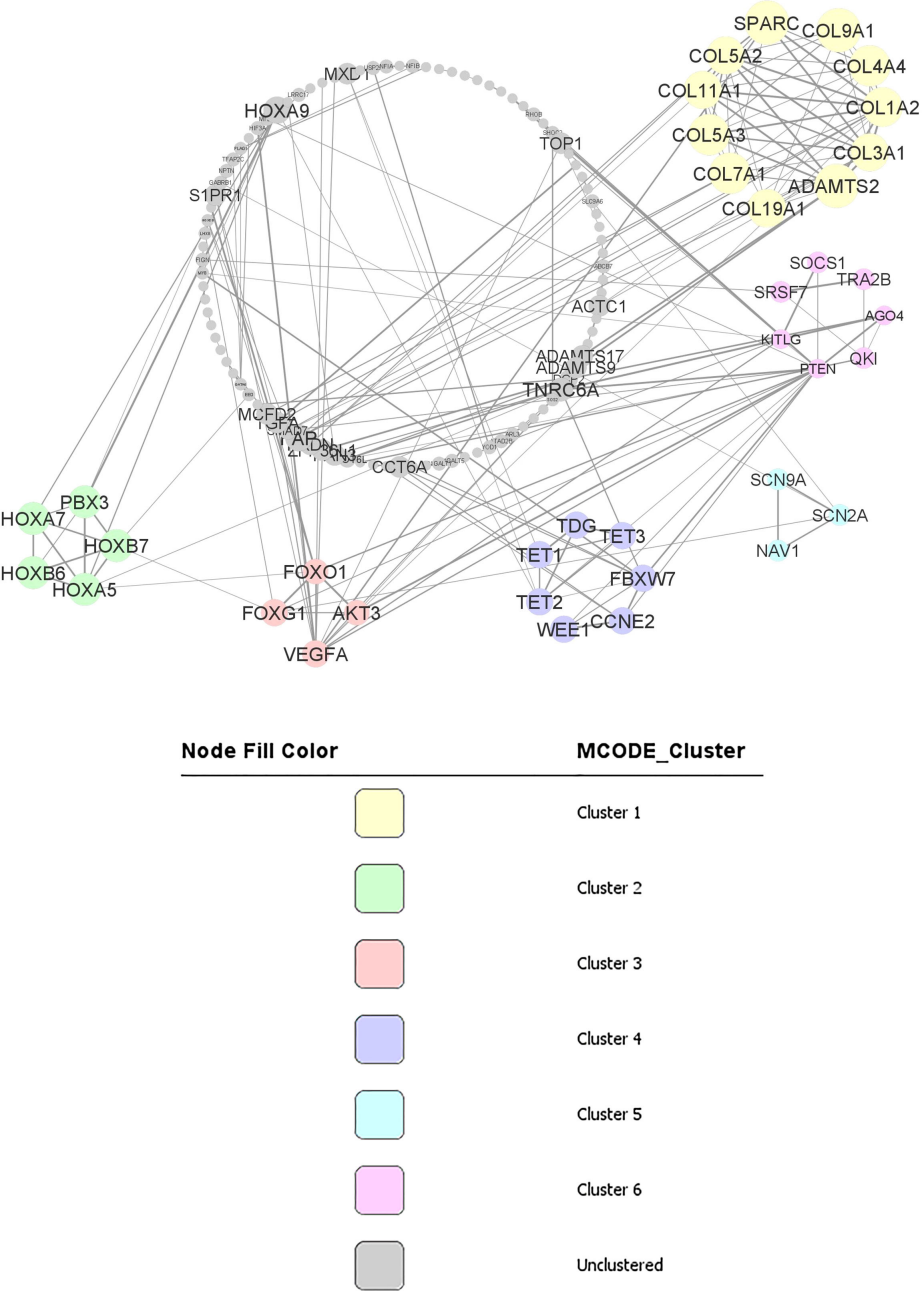
PPI network and modular analysis of downstream genes. From STRING online database analysis, a total of 187 proteins were filtered into a PPI network complex with 136 nodes and 211 edges. Six clusters were identified from Cytoscape MCODE.

Using STRING online database (http://string-db.org), a total of 187 proteins from predicted target genes were filtered into a PPI network complex, containing 136 nodes and 211 edges (Figure 3) with PPI enrichment p-value is less than 1.0E-16. At the same time, 51 other proteins did not fall into the PPI network.

The results were transferred from STRING to Cytoscape for further analysis. Through Cytoscape MCODE, six significant modules from the PPI network complex were found. Functional annotation clustering showed that cluster 1 (score = 9) consisted of 11 nodes and 45 edges (**Figure 3**). Cluster1 is mainly located in the extracellular region and associated with extracellular matrix organisation and the collagen catabolic process. Cluster 2 (score= 5) consisted of five nodes and ten edges (**Figure 3**) associated with homeobox, sequence-specific DNA binding and transcription regulation. Cluster 3 (score= 3.333) consisted of four nodes and five edges (**Figure 3**) associated with the FoxO signalling pathway, pathways in cancer and mutagenesis site. Cluster 4 (score= 3.33) consisted of seven nodes and ten edges (**Figure 3**) associated with polymorphism, nucleus and DNA methylation. Cluster 5 (score= 3) consisted of three nodes and five edges (**Figure 3**) associated with coiled-coil structure and sodium ion transport channel. Cluster 6 (2.667) consisted of seven nodes and eight edges (**Figure 3**) located in the cytoplasm and nucleus and associated with protein binding, RNA binding and phosphatidylinositol-mediated signalling. **Table 7** includes functional annotation clustering for all six clusters. The list of genes ontology of cluster 1 until 6 are included in ‘**Data S7**’.

**Table 7:** Functional annotation clustering of cluster 1 to 6.

Cluster	Term	Description	Count	p-value
1	Up_Keywords: Extracellular matrix	Extracellular matrix	11	8.05E-20
	CC_ GO:0005576	Extracellular region	11	2.82E-11
	Up_Keywords: Secreted	Secreted	11	6.17E-11
	Up_Keywords: Disulfide bond	Disulfide bond	11	1.65E-08
	Up_Keywords: Signal	Signal	11	1.13E-07
	Up_Keywords: Polymorphism	Polymorphism	11	0.004699
2	Up_Keywords:	Homeobox	5	2.57E-08
	MF_GO:0043565	Sequence-specific DNA binding	5	8.77E-07
	Up_Keywords: DNA-binding	DNA-binding	5	9.82E-05
	Up_Keywords: Transcription regulation	Transcription regulation	5	1.64E-04
	Up_Keywords: Transcription	Transcription	5	1.84E-04
	Up_Keywords: Nucleus	Nucleus	5	0.004211
	CC_GO:0005634	Nucleus	5	0.007789
3	hsa04068	FoxO signaling pathway	3	0.001116
	hsa05200	Pathways in cancer	3	0.009398
4	Up_Keywords: Polymorphism	Polymorphism	7	0.040122
	Up_Keywords: Nucleus	Nucleus	6	0.005069
	GO:0005634	nucleus	6	0.010445
5	Up_Keywords: Coiled coil	Coiled coil	3	0.021754
	GO:0005248	voltage-gated sodium channel activity	2	0.001185
	GO:0001518	voltage-gated sodium channel complex	2	0.001536
6	MF_ GO:0005515	protein binding	7	0.019848
	CC_ GO:0005737	cytoplasm	6	0.008813
	CC_ GO:0005634	nucleus	6	0.010445

## Discussion

**Figure 4:**
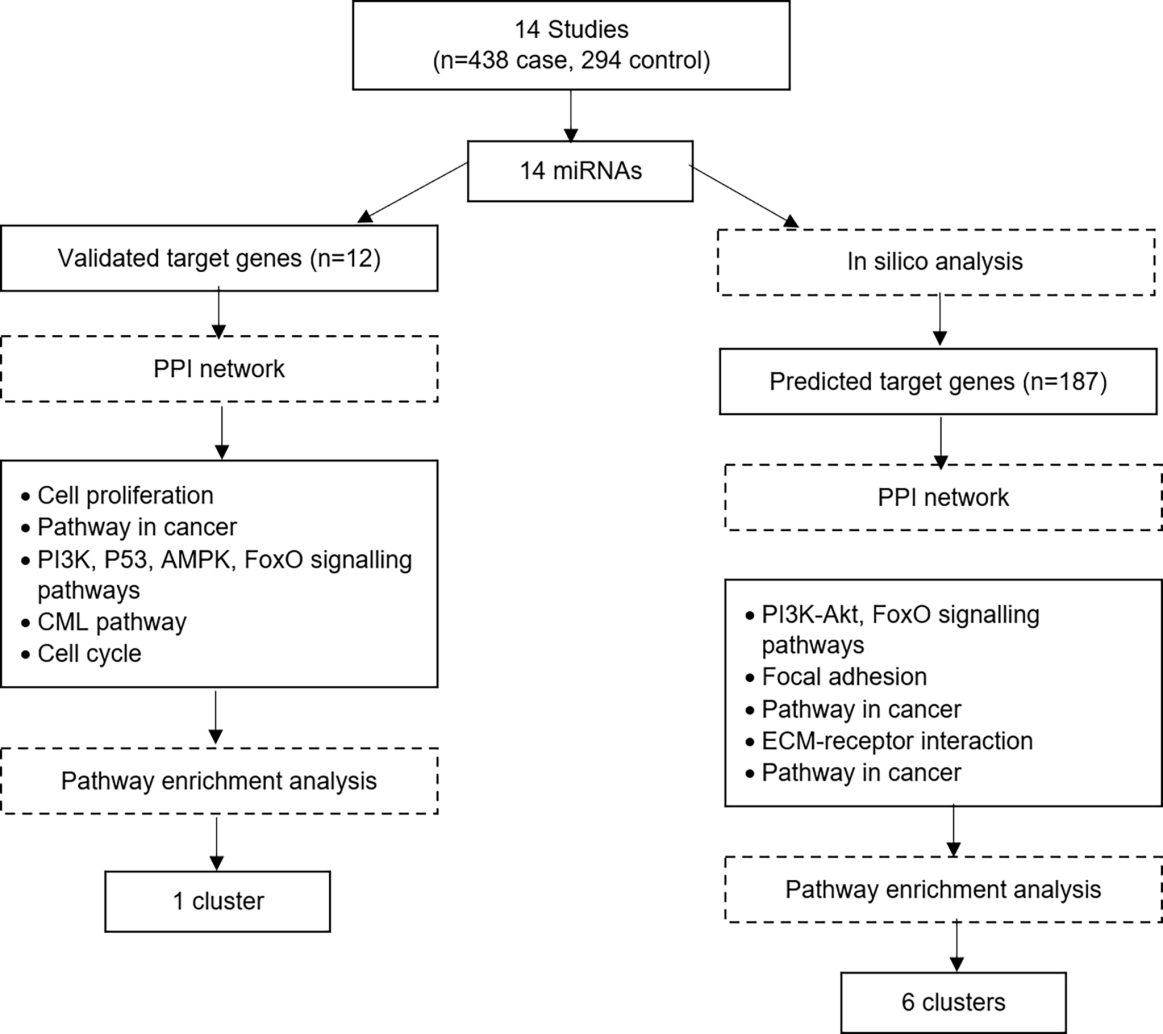
Summary of the bioinformatical analysis findings.

Tyrosine kinase inhibitors that target BCR-ABL protein have been successfully used to treat CML. However, there are still many cases that does not response well to the treatment. In those cases, it is postulated that their CML pathogenesis does not only involves BCR-ABL oncogene, it involves other mechanisms. For many decades, numerous molecular and clinical studies involving chromosomal changes, DNA mutation, DNA methylation and mRNA expression have been done to understand the underlying mechanism of CML development and progression. Nevertheless, the complete mechanisms of CML remain unclear. Thus, since many years ago, miRNA has come into the picture and has been studied extensively since then. In this systematic review, we attempted to improve our understanding of the involvement of miRNAs in CML development from fourteen different reports. The studies’ dataset was divided into two datasets to make sure high coverage of target genes. **Figure 4** shows the summary of the bioinformatical analysis findings. The analysis of the first dataset started with pooling of the miRNA and their validated downstream genes, followed by gene’s ontology analysis, signalling pathway enrichment analysis and finally, the protein-protein interaction network and modular analysis. One cluster was revealed from the first dataset. However, a second dataset was constructed and analysed to have a broader view of miRNA involvement in CML pathogenesis. The analysis of the second dataset involved the pooling of the miRNAs, followed by prediction of their target genes, gene’s ontology analysis, signalling pathway enrichment analysis and finally, the protein-protein interaction network and modular analysis. Six significant clusters were revealed from the second dataset.

In this review, through integrated bioinformatical analysis, a cluster consisted of 12 nodes and 49 edges has been identified from the first dataset consisting of the reported target genes and downstream genes. All the 12 clustered proteins are enriched in the intracellular signalling through phosphorylation, and are involved in cancer pathogenesis. Seven of these proteins (MAPK8, CCND1, IRS1, MYC, BCL2, BAX, AKT1) are enriched in the cytosol and nucleus. Three proteins (PIK3CA, IRS2, BCL2L1) are enriched in the cytosol, and one protein is in the nucleus (GADD45A). MAPK8, CCND1, IRS1, GADD45A, MYC, BCL2, AKT1, IRS2, and BCL2L1 are phosphoproteins. Phosphoproteins are proteins that bind to the phosphate group that can be switched on or off. These proteins are essential in most cellular processes such as protein synthesis, cell division, signal transduction, cell growth, development, and ageing (32).

From the KEGG pathway, MAPK8, CCND1, PIK3CA, MYC, BCL2, BAX, AKT1, MMP9, and BCL2L1, are essential genes in cancer pathways that regulate cell cycle, proliferation, apoptosis, genomic instability, and block of differentiation. At the same time, MAPK8, CCND1, PIK3CA, IRS1, GADD45A, AKT1 and IRS2 are involved in FoxO signalling pathway that can affect the regulation of cell cycle, oxidative stress resistance and DNA repair.

The first dataset of those experimentally validated target genes shows a significant effect of miRNAs in CML pathogenesis. However, it is known that miRNA can target more than one target gene due to its seed sequence (33). Therefore, it is crucial to identify the other miRNA’s target genes and their pathways involved. This review identified the other miRNA’s target genes using four different in silico analyses. All the predicted target genes were then analysed using bioinformatical analysis. Six clusters were identified from the functional annotation clustering analysis of 187 proteins.

Cluster 1 from the second dataset consisted of 11 proteins, including COL3A1, ADAMTS2, SPARC, COL1A2, COL11A1, COL4A4, COL5A3, COL7A1, COL5A2, COL9A1, COL19A1. All these proteins are in the extracellular region and appear to be crucial for extracellular matrix organization. COL3A1, COL1A2, COL11A1, COL4A4, COL5A3, and COL5A2 are involved in ECM-receptor interaction pathways and focal adhesion pathways. This collagen genes family is essential as the component of tissues structure and can interact with cells *via* several receptor families and regulate cell’s proliferation, differentiation and migration (34). Other than that, SPARC, another gene in cluster 1, is significantly downregulated in CML patients. In CML cells exposed to exogenous SPARC, the G0/G1 cell cycle arrest and reduced growth rate of the cells were reported (35). However, the mechanism involved in SPARC downregulation in CML is still unknown. Thus, in CML, inhibition of SPARC by miR-29b-3p could be one of the CML pathogenesis mechanisms and is worth investigating.

Cluster 2 consisted of five proteins, including PBX3, HOXA7, HOXB7, HOXB6, and HOXA5, enriched in the nucleus. These proteins have sequence-specific DNA binding and are essential in transcription regulation. Interestingly, PBX3 is an important co-factor for the HOXA gene family (36). The HOX family is a group of highly conserved genes in mammals and are crucial in regulating cell differentiation and proliferation (36). HOXA5 impairs myelopoiesis, causing blockage of hematopoietic stem cells differentiation. The downregulation of HOXA5 is commonly related to DNA methylation (37). Apart from DNA methylation, the current review also found that miR-196b-5p could also regulate HOXA5 expression in CML cells.

Cluster 3 consisted of FOXG1, AKT3, VEGFA dan FOXO1. From KEGG pathway analysis, FOXG1, AKT3, and FOXO1 are enriched in the FoxO signalling pathway, while AKT3, FOXO1, and VEGFA are important in the cancer pathway having essential roles in apoptosis, proliferation and angiogenesis. FOXO1 is a Forkhead box O (FoxO) family member. It plays a role in the regulation of differentiation and metabolism in tissues and organs. In CML, FOXO1 could increase the activation of CML cells (38). Thus, it will be an excellent move to inhibit FOXO1 in deactivating CML cells. In this review, it is suggested that miR-223-3p is a potential regulator of FOXO1 in CML.

Cluster 4 consisted of seven proteins, including WEE1, CCNE2, FBXW7, TDG, TET3, TET2, and TET1. Six proteins (WEE1, CCNE2, TDG, TET3, TET2, and TET1) are located in the nucleus, while four (TDG, TET3, TET2, TET1) are involved in DNA methylation. TET oncogene family that includes TET1, TET2, and TET3 plays a role in the DNA methylation process (39). Although there are no reports on the role of TET genes in CML pathogenesis, TET3 has been identified as a prognostic biomarker for acute myeloid leukaemia (AML) (40), suggesting its involvement in myeloproliferative pathogenesis. Furthermore, the WEE1 gene in the cluster has been linked to the cell cycle and identified as a critical mediator of cell fate in AML (41). Meanwhile, high WEE1 kinase expression in acute lymphoblastic leukaemia (ALL) has been identified as a poor prognostic factor that functions as a cancer-conserving oncogene which helps protect cancer cells from DNA damage (42). As a result of WEE1’s participation in the cell cycle and DNA damage repair, the WEE1 kinase family was identified as one of the most promising targets in the DNA damage response (DDR) pathway (43).

Cluster 5 consisted of three proteins, including SCN9A, NAV1, and SCN2A. These proteins are involved in the voltage-gated sodium channel complex. Although there are still no CML studies on these genes, ion channel signalling mechanisms are known to be involved in cancer cells migration, invasion, and metastasis (44). Thus, it will be very informative to study the effect of ion channel signalling through SCN9A and SCN2A *via* miR-301-5p in CML cells.

Cluster 6 consisted of seven protein-binding proteins, including KITLG, SOCS1, AGO4, TRA2B, PTEN, SRSF7, and QKI. KITLG is localised in the cytoplasm, TRA2B is in the nucleus, while SOCS1, AGO4, PTEN, SRSF7, and QKI can be found in both intracellular regions. KITLG, AGO4, PTEN are involved in phosphatidylinositol-mediated signaling that is crucial in regulating cancer cells’ survival, proliferation, invasion, and growth. SOCS1 plays an important role in regulating optimal JAK/STAT activity. However, regulation of SOCS1 *via* DNA methylation in CML patients is still uncertain as the findings are contradictory (45, 46). Thus, from the analysis, regulation of SOCS1 *via* miR-30a-5p in CML is suggested.

The studies included in this review only focused on their miRNA of interest and its few target genes. Therefore, this review are not able to rule out the entire networks of miRNAs and their target genes in CML. In silico analyses were done to improve the coverage of miRNA’s target genes. However, further *in vitro* analysis and clinical studies need to be done to validate the predicted mechanisms. Nevertheless, this review added new insight into the involvement of miRNA in CML pathogenesis for future studies.

## Conclusion

Pathogenesis of CML at the molecular level involves a wide range of mechanisms that are still undiscovered. In this study, the function of miRNAs was found to be significant in the development of CML. The miRNA’s target genes are localised in the extracellular, cytosol and nucleus of CML cells. Thus, the importance of miRNAs cannot be denied as miRNAs are universally involved in various pathways that regulate genomic instability, proliferation, apoptosis, cell cycle, differentiation, and migration of CML cells. Therefore, from the identified miRNAs and their pathways involved in CML pathogenesis, potential new biomarkers for a better prognosis and new miRNA-based treatment for CML patients could be developed.

## Data Availability Statement

The original contributions presented in the study are included in the article/**Supplementary Material**. Further inquiries can be directed to the corresponding author.

## Author Contributions

ME collected and/or assembled data and wrote the manuscript. SS wrote, reviewed, and edited the manuscript. NA confirm the authenticity of all raw data, proofread, edited and gave approval of the manuscript. All authors contributed to the article and approved the submitted version.

## Funding

This study was funded by the Fundamental Research Grant Scheme, awarded by Ministry of Higher Education of Malaysia, grant number FRGS/1/2018/SKK08/USIM/03/1.

## Conflict of Interest

The authors declare that the research was conducted in the absence of any commercial or financial relationships that could be construed as a potential conflict of interest.

## Publisher’s Note

All claims expressed in this article are solely those of the authors and do not necessarily represent those of their affiliated organizations, or those of the publisher, the editors and the reviewers. Any product that may be evaluated in this article, or claim that may be made by its manufacturer, is not guaranteed or endorsed by the publisher.
